# Reconstructions of the dark-energy equation of state and the inflationary potential

**DOI:** 10.1007/s10714-018-2402-4

**Published:** 2018-06-19

**Authors:** John D. Barrow, Andronikos Paliathanasis

**Affiliations:** 1 DAMTP, Centre for Mathematical Sciences, University of Cambridge, Wilberforce Rd., Cambridge, CB3 0WA UK; 2 Instituto de Ciencias Físicas y Matemáticas, Universidad Austral de Chile, Valdivia, Chile; 3 Department of Mathematics and Natural Sciences, Core Curriculum Program, Prince Mohammad Bin Fahd University, Al Khobar, 31952 Kingdom of Saudi Arabia; 4 Institute of Systems Science, Durban University of Technology, PO Box 1334, Durban, 4000 South Africa

**Keywords:** Cosmology, Scalar field, Inflation

## Abstract

We use a mathematical approach based on the constraints systems in order to reconstruct the equation of state and the inflationary potential for the inflaton field from the observed spectral indices for the density perturbations $n_{s}$ and the tensor to scalar ratio *r*. From the astronomical data, we can observe that the measured values of these two indices lie on a two-dimensional surface. We express these indices in terms of the Hubble slow-roll parameters and we assume that $n_{s}-1=h\left( r\right)$. For the function $h\left( r\right)$, we consider three cases, where $h\left( r\right)$ is constant, linear and quadratic, respectively. From this, we derive second-order equations whose solutions provide us with the explicit forms for the expansion scale-factor, the scalar-field potential, and the effective equation of state for the scalar field. Finally, we show that for there exist mappings which transform one cosmological solution to another and allow new solutions to be generated from existing ones.

## Introduction

An ‘inflaton’ is a scalar field that can drive a period of acceleration in the early universe. Such a finite period of inflation [1, 2] can solve long-standing problems about the structure of the universe that would otherwise require special initial conditions [3, 4]. An inflaton provides a matter source that can display antigravitating behavior and so it could also be a candidate for the so-called the ‘dark energy’ that drives cosmological acceleration today. It is possible that these two eras of cosmological acceleration are connected, but so far there is no compelling theory about how that link might arise between two such widely separated energy scales.

Various inflationary self-interaction potentials for the inflaton have been proposed in the literature. Since they lead to different inflationary scenarios, particularly in respect of the density fluctuations produced, they have different observational consequences for the cosmic microwave background radiation, and this permits them to be finely constrained by observational data. Various inflaton potentials in general relativistic scalar field cosmology have been proposed in [5–18] , while for inflationary models in other gravity theories, where there are more possibilities, see [1, 19–30] and references therein.

The construction of the inflaton scalar field potential from observational data is an open problem of special interest. It provides critical information about the details of the allowed inflationary models and might provide clues as to the identity of the inflaton. In [31–37], the perturbative reconstruction approach was applied: the inflaton self-interaction potential, $V\left( \phi \right)$, of the scalar field, $\phi$, was reconstructed by considering a series expansion around a point $\phi =\phi _{0}$, where the coefficients of the series expansion for the potentials are determined from the observable values of the scalar spectral index and the usual slow-roll parameters; for more details see [38]. Alternative approaches to the reconstruction of the scalar field potential include a stochastic perturbative approach in [39], or another perturbative approach in [40]. Two alternative methods for the reconstruction of the scalar field potential have been proposed in [41] and [42]. Specifically, in the latter work, an exponential of the scalar field’s Hubble function was considered and found to offer an efficient way to derive and constrain the power-spectrum observables [42]. By contrast, in [41], the scalar field potential was reconstructed for the Harrison–Zeldovich spectrum by solving the gravitational field equations along with the equation for the adiabatic scalar perturbations.

The slow-roll parameters and their relations to the spectral indices have been reconstructed in closed-form [43–45]. This is the approach that we will follow here to find the equation of state for the effective perfect fluid which corresponds to the scalar field with a self-interaction potential. While this approach is not so accurate as the previous approaches (because it depends on approximate relations between the spectral indices and the slow-roll parameters [38]) it can more easily reconstruct closed-form solutions for the inflationary potential and the expansion scale factor expansion. Furthermore, as we shall see in the first approximations for the models that we study, there exist mappings which transform the models to other equivalent models and their linearised fluctuations to the Harrison–Zeldovich spectrum. The plan of this paper is as follows.

In Sect. 2, we review scalar field cosmology in a spatially flat Friedmann–Lemaître–Robertson–Walker (FLRW) universe and introduce the basic quantities and notations. In Sect. 3, we assume that the spectral index for the density perturbations, $n_{s}$, and the tensor-to-scalar ratio, *r*, are related by a function such that $n_{s}-1=h\left( r\right)$. For the defining function, $h\left( r\right) ,$ we assume that it is either constant, linear or quadratic in *r*. Moreover, using the slow-roll expressions for these indices, we find ordinary differential equations whose solutions provide us with the inflationary scalar field potentials and the equation of state for the energy density and the pressure of the scalar field while the density perturbations to tensor-to-scalar ration diagrams are presented for the analytical solutions that we derive. Moreover, in Sect. 4 the values for the free parameters of the models are determined in order a late time attractor to exists such that the universe to escape from the inflation phase. Moreover, a transformation which relates the different models that we study is presented in Sect. 5. We show that our master equations are all maximally symmetric. This ensures that maps exist which can transform the solution of one inflationary model into another. This can be used to determine new inflationary solutions from known ones. A discussion of the results presented and our conclusions are given in the concluding Sect. 6.

## Underlying equations and definitions

We take the gravitational field equations to be (with units $8\pi G=c=\hslash =1$)

1 $$\begin{aligned} G_{\mu \nu }=T_{\mu \nu }^{\left( \phi \right) }+T_{\mu \nu }^{\left( m\right) }, \end{aligned}$$

where $G_{\mu \nu }= R_{\mu \nu }-\frac{1}{2}g_{\mu \nu }R$ is the Einstein tensor, $T_{\mu \nu }^{\left( \phi \right) }$ is the energy-momentum tensor for the scalar field,

2 $$\begin{aligned} T_{\mu \nu }^{\left( \phi \right) }=\phi _{;\mu }\phi _{;\nu }-g_{\mu \nu }\left( \frac{1}{2}\phi ^{;\sigma }\phi _{;\sigma }-V\left( \phi \right) \right) , \end{aligned}$$

and $T_{\mu \nu }^{\left( m\right) }$ denotes the energy-momentum tensor of the other matter sources. Now, we will assume that the universe contains only the scalar field, so $T_{\mu \nu }^{\left( m\right) }=0$. In addition, we have the propagation equation for the scalar field, $\phi$, from the Bianchi identity $T_{~~~~~~~\ ;\nu }^{\left( \phi \right) \mu \nu }=0,$ which is

3 $$\begin{aligned} -g^{\mu \nu }\phi _{;\mu \nu }+V_{,\phi }=0. \end{aligned}$$

For a spatially-flat FLRW universe, with scale factor, $a\left( t\right)$, the field equations (1–3) are

4 $$\begin{aligned} 3H^{2}= & {} \frac{1}{2}{\dot{\phi }}^{2}+V(\phi ), \end{aligned}$$

5 $$\begin{aligned} 2{\dot{H}}+3H^{2}= & {} -\frac{1}{2}{\dot{\phi }}^{2}+V(\phi ), \end{aligned}$$

and

6 $$\begin{aligned} {\ddot{\phi }}+3H{\dot{\phi }}+V_{,\phi }=0, \end{aligned}$$

where $H=\frac{{\dot{a}}}{a}$ is the Hubble function and overdots denote differentials with respect to comoving proper time, *t*. The comoving observers have $u^{\mu }=\delta _{0}^{\mu }$ , so $u^{\mu }u_{\mu }=-1$. The FLRW symmetries ensure $\phi =\phi \left( t\right)$.

From (2), we find that the energy density of the scalar field for the comoving observer is

7 $$\begin{aligned} \rho _{\phi }\equiv \frac{1}{2}{\dot{\phi }}^{2}+V(\phi ); \end{aligned}$$

the pressure is $P_{\phi }=w_{\phi }\rho _{\phi }$, where $w_{\phi }$ is the equation of state parameter (EoS):

8 $$\begin{aligned} w_{\phi }=\frac{{\dot{\phi }}^{2}-2V(\phi )}{{\dot{\phi }}^{2}+2V(\phi )}. \end{aligned}$$

The deceleration parameter, *q*, is given by the formula $q=\frac{1}{2}\left( 1+3w_{\phi }\right)$ because, as the only matter source is the scalar field, we have $w_{tot}=w_{\phi }$. The expansion of the universe is accelerated when $q<0$, that is, $w_{\phi }<-\frac{1}{3}$. Since $V\left( \phi \right) >0$, a negative negative EoS parameter means that the potential dominates the kinetic term i.e., $\frac{{\dot{\phi }}^{2}}{2}<V(\phi )$. Furthermore, in the limit ${\dot{\phi }}\rightarrow 0$ expression (8) gives $w_{\phi } \rightarrow -1$, and the scalar field mimics the cosmological constant.

The so-called potential slow-roll parameters (PSR),

9 $$\begin{aligned} \varepsilon _{V}=\left( \frac{V_{,\phi }}{2V}\right) ^{2},\quad \eta _{V} =\frac{V_{,\phi \phi }}{2V}, \end{aligned}$$

have been introduced [46] in order to study the existence of the inflationary phase of the universe. Specifically, the condition for an inflationary universe is $\varepsilon _{V}<<1$, while in order for the inflationary phase to last long enough we require the second PSR parameter also to be small, $\eta _{V}<<1$.

Similarly, the Hubble slow-roll parameters (HSR) have been defined by [47, 48]      

10 $$\begin{aligned} \varepsilon _{H}=-\frac{d\ln H}{d\ln a}=\left( \frac{H_{,\phi }}{H}\right) ^{2}, \end{aligned}$$

and

11 $$\begin{aligned} \eta _{H}=-\frac{d\ln H_{,\phi }}{d\ln a}=\frac{H_{,\phi \phi }}{H}. \end{aligned}$$

It has been shown that the HSR slow-roll parameters are more accurate descriptors of inflation than the PSR parameters. However, the PSR and HSR parameters are related and, when $\varepsilon _{H}$ and $\eta _{H}$ are small, these relations become

12 $$\begin{aligned} \varepsilon _{V}\simeq \varepsilon _{H}~\text {and~}\eta _{V}\simeq \varepsilon _{H}+\eta _{H}. \end{aligned}$$

In the following we choose to work with the HSR parameters.

### Analytical solution

Recently, in ref. [49], it was found that the field equations, (4–6), under the transformation, $dt=\exp \left( \frac{F\left( \omega \right) }{2}\right) d\omega$ with $\omega =6\ln a,$ where *a*(*t*) is the cosmic scale factor, can be solved for the scalar field, $\phi$, and the potential, $V\left( \phi \right) ,$ by the following formulae^1^

13 $$\begin{aligned} \phi (\omega )=\pm \frac{\sqrt{6}}{6}\int \!\!\sqrt{F^{\prime }(\omega )} d\omega \end{aligned}$$

and

14 $$\begin{aligned} V(\omega )=\frac{1}{12}e^{-F(\omega )}\left( 1-F^{\prime }(\omega )\right) , \end{aligned}$$

so the line-element for the FLRW spacetime is now

15 $$\begin{aligned} ds^{2}=-e^{F\left( \omega \right) }d\omega ^{2}+e^{\omega /3}(dx^{2} +dy^{2}+dz^{2}). \end{aligned}$$

For the latter line element, the Hubble function is defined as$~H\left( \omega \right) =\frac{1}{6}e^{-\frac{F}{2}}$, from where with the use of (13) it follows $\frac{dH}{d\phi }=\pm \frac{\sqrt{6}}{2}e^{-\frac{F}{2}}F^{\prime },$ then expression (15) reduces to the Hamilton-Jacobi like equation, for $H\left( \phi \right)$,

$$\begin{aligned} 2\left( \frac{dH\left( \phi \right) }{d\phi }\right) ^{2}-3\left( H\left( \phi \right) \right) ^{2}+V\left( \phi \right) =0, \end{aligned}$$

which studied in [50, 51].

In the new variables, the effective fluid components for the scalar field are

16 $$\begin{aligned} \rho _{\phi }(\omega )=\frac{1}{12}e^{-F(\omega )},\quad P_{\phi }(\omega )=\frac{1}{12}e^{-F(\omega )}\left( 2F^{\prime }(\omega )-1\right) , \end{aligned}$$

and the effective EoS parameter takes the simple form

17 $$\begin{aligned} w_{\phi }\left( \omega \right) =\left( 2F^{\prime }(\omega )-1\right) . \end{aligned}$$

These expressions hold for an arbitrary scalar field potential. The field equations have been reduced to a single first-order differential equation which can be viewed as a form of the equation of state, $P_{\phi }=P_{\phi }\left( \rho _{\phi }\right)$, for the scalar field. This approach was applied in [52] in order to construct inflationary potentials from specific linear and non-linear equations of state.

We can use this solution to express the slow-roll parameters, PSR or HSR, in terms of the new variable $\omega \equiv \ln (a^{6})$. The HSR parameters are found to be [52]

18 $$\begin{aligned} \varepsilon _{H}=3F^{\prime },\quad \eta _{H}=3\frac{\left( F^{\prime }\right) ^{2}-F^{\prime \prime }}{F^{\prime }}, \end{aligned}$$

or, equivalently in terms of the effective EoS parameter,

19 $$\begin{aligned} \varepsilon _{H}=\frac{3}{2}\left( 1+w_{\phi }\left( \omega \right) \right) , \end{aligned}$$

and

20 $$\begin{aligned} \eta _{H}=\frac{3}{2}\frac{\left( w_{\phi }+1\right) ^{2}-2w_{\phi ,\omega } }{\left( 1+w_{\phi }\right) }. \end{aligned}$$

The number of e-folds is defined to be $N_{e}=\int _{t_{i}}^{t_{f}}H\left( t\right) dt=\ln \frac{a_{f}}{a_{i}}=\frac{1}{6}\left( \omega _{f}-\omega _{i}\right) ,$ which means that $N_{e}$ is linearly related to the function $\omega$. Hence, the slow-roll parameters can be expressed in terms of $N_{e}$.

Lastly, using expression (19), all the slow-roll parameters can be expressed in terms of the parameter $\varepsilon _{H}$ and its derivatives.

## Reconstruction of the inflationary potential

From the recent data analysis by the Planck 2015 collaboration [4], the value of the spectral index for the density perturbations is $n_{s}=0.968\pm 0.006,~$while the range of the scalar spectral index is $n_{s}^{\prime }=-0.003\pm 0.007$. The tensor to scalar ratio, *r*, has a value smaller than 0.11, i.e., $r<0.11$.

The mathematical expression which relates the HSR parameters to the spectral indices $n_{s}$ in the first approximation is

21 $$\begin{aligned} n_{s}\equiv 1-4\varepsilon _{H}+2\eta _{H}, \end{aligned}$$

while the tensor to scalar ratio is $r=10\varepsilon _{H}$. Moreover, in the second approximation the spectral index, $n_{s}$, becomes

22 $$\begin{aligned} n_{s}\equiv 1-4\varepsilon _{H}+2\varepsilon _{H}-8\left( \varepsilon _{H}\right) ^{2}\left( 1+2C\right) +\varepsilon _{H}\eta _{H}\left( 10C+6\right) -2C\xi _{H}, \end{aligned}$$

where $C=\gamma _{E}+\ln 2-2=-0.7296$. So, now it follows that the running index is

23 $$\begin{aligned} n_{s}^{\prime }\equiv 2\varepsilon _{H}\eta _{H}-2\xi _{H}. \end{aligned}$$

From the analysis of the previous section, the spectral indices for the FLRW spacetime can be written in terms of $\varepsilon _{H}$ and its derivative, or in terms of the unknown function, $F\left( \omega \right)$, and its derivatives. Recall, that the above expressions for the spectral indices are definitions and not deductions. However, if we assume that the left-hand side satisfies some functional expression, i.e., $n_{s}=h\left( \varepsilon _{H},\ldots \right)$, for an function *h*, then we define a differential equation, which can be used to construct the exact form for the FLRW spacetime (15), i.e., determine $F\left( \omega \right)$, that satisfies the spectral index conditions. Hence, with the use of the solution presented in the previous section, the scalar field potential can also be derived.

In the following, we consider that

24 $$\begin{aligned} n_{s}-1=h\left( r\right) , \end{aligned}$$

and we work with the expression (21) in the first-order approximation. Moreover, we assume that we are close to the $n_{s}=1$ spectrum so that we can treat $h\left( r\right)$ as a small correction term to the spectrum. Hence, the Taylor expansion of the $h\left( r\right)$ function close to a constant value for the scalar ratio, that is, $r=r_{0}$, yields

25 $$\begin{aligned} h\left( r\right) =h\left( r_{0}\right) +h^{\prime }\left( r_{0}\right) \left( r-r_{0}\right) +\frac{h^{\prime \prime }\left( r_{0}\right) }{2!}\left( r-r_{0}\right) ^{2}+\cdots \end{aligned}$$

For our analysis we select three forms for the function *h*(*r*),  which include the three first terms of the last Taylor expansion for the function $h\left( r\right)$. Hence, by substituting from (18) in (24) three master equations follow for each chosen form of $h\left( r\right)$.

### Constant index: $n_{s}-1=-2n_{0}$

Assume that the spectral index for the density perturbations is constant, with $n_{s}-1=-2n_{0}$, where according to the Planck 2015 data at $1\sigma$, $n_{0}$ should be bounded in the range $\,0.013\le n_{0}\le 0.019$. In the case where $n_{0}=0$, i.e., $n_{s}=1$, we have the Harrison–Zeldovich spectrum. These cases were studied before in [43–45].

#### Zero $n_{0}:~$Harrison–Zeldovich spectrum

Let $n_{0}=0$, so $n_{s}=1~$and we have the exact Harrison–Zeldovich spectrum. Then, from (21), it follows that $\eta _{H}=2\varepsilon _{H}$. Hence, from (18) the second-order differential equation for $F(\omega )$ is

26 $$\begin{aligned} F^{\prime \prime }+\left( F^{\prime }\right) ^{2}=0, \end{aligned}$$

which has the solution $F\left( \omega \right) =\ln \left( F_{1}\left( \omega -\omega _{0}\right) \right)$, where the effective equation-of-state parameter is now

27 $$\begin{aligned} w_{\phi }\left( \omega \right) =-1+\frac{2}{\omega -\omega _{0}}. \end{aligned}$$

The differential equation, (26), was derived in [52] and it follows from the generalized Chaplygin gas [56] with $\lambda =2$, that is for an EoS

28 $$\begin{aligned} p_{\phi }=\gamma \rho _{\phi }^{\lambda }-\rho _{\phi }~\text {with}~\lambda =2, \end{aligned}$$

where $\gamma \varpropto F_{1}$. Therefore, with the use of expressions (13–15) we find that in the proper time where $N\left( t\right) =1$, the scale factor is that of an intermediate inflation ([41–56]),

29 $$\begin{aligned} a\left( t\right) \simeq \exp \left( a_{1}t^{2/3}\right) , \end{aligned}$$

and the scalar-field potential is

30 $$\begin{aligned} V\left( \phi \right) =\frac{1}{18F_{1}}\left( \phi ^{-2}-\frac{2}{3}\phi ^{-4}\right) . \end{aligned}$$

Here it is important to mention that the scalar field description of the inflaton is valid only for values of $\phi$, such that the scalar field potential is not negative. Note that the non essential integration constants have been absorb and $\phi$ indicates $\phi -\phi _{0}$, where in (30) without loss of generality we considered $\phi _{0}=0$.

#### Non-zero $n_{0}$

We now assume that $n_{s}-1=-2n_{0}\ne 0$. Then, from (21), it follows that $\eta _{H}=2\varepsilon _{H}-n_{0}$ and with the use of (18), the differential equation for $F(\omega )$ is now

31 $$\begin{aligned} F^{\prime \prime }+\left( F^{\prime }\right) ^{2}-\frac{n_{0}}{3}F^{\prime }=0, \end{aligned}$$

with the closed-form solution

32 $$\begin{aligned} F\left( \omega \right) =\ln \left\{ F_{1}\exp \left( \frac{n_{0}}{3} \omega \right) \right\} +F_{0}. \end{aligned}$$

The latter function has been derived in [56] as the solution in which the scalar field mimics the generalized Chaplygin gas (or a bulk viscosity) with EoS parameter

33 $$\begin{aligned} p_{\phi }=A\rho _{\phi }^{2}+B\rho _{\phi } \end{aligned}$$

for the specific values *A*, *B* such that $F_{0}=-\frac{A}{1+B}$ and $B=1+\frac{2}{3}n_{0}$.

Furthermore, the effective EoS parameter is calculated to be

34 $$\begin{aligned} w_{\phi }\left( \omega \right) =-1+\frac{n_{0}}{3}-\frac{F_{0}n_{0}}{3}\left( F_{1}\exp \left( \frac{n_{0}}{3}\omega \right) +F_{0}\right) ^{-1}, \end{aligned}$$

while the closed-form expression for the scalar field potential is

35 $$\begin{aligned} V\left( \phi \right) =\frac{F_{1}}{9}\frac{e^{\sqrt{\frac{n_{0}}{3}}\phi } }{\left( e^{\sqrt{\frac{n_{0}}{3}}\phi }+F_{0}F_{1}\right) ^{2}}\left( 3-n_{0}\left( \frac{e^{\sqrt{\frac{n_{0}}{3}}\phi }-F_{0}F_{1}}{e^{\sqrt{\frac{n_{0}}{3}}\phi }+F_{0}F_{1}}\right) ^{2}\right) . \end{aligned}$$

The expansion scale factor cannot be written in a closed-form expression in the proper time, *t*. Moreover, for the potential (35), we have that for large values of $\phi$, the potential becomes exponential, that is,

36 $$\begin{aligned} \lim _{\phi \rightarrow +\infty }V\left( \phi \right) =\frac{\left( 3-n_{0}\right) F_{1}}{9}e^{-\sqrt{\frac{n_{0}}{3}}\phi }, \end{aligned}$$

and approximates the solution in which the scalar field mimics a perfect fluid with constant equation of state parameter. In order to determine the physical properties of the parameter $n_{0}$, but also those of the integration constants $F_{0}$ and $F_{1}$, the indices $n_{s}$ and *r* are calculated below.

#### Observational constraints

For the solution (32), we calculate the slow-roll parameters to be

37 $$\begin{aligned} \varepsilon _{H}=n_{0}\left( 1-\left( 1+\frac{F_{1}}{F_{0}}e^{\frac{n_{0}}{3}\omega }\right) ^{-1}\right) ,\quad \eta _{H}=2\varepsilon _{H}-n_{0}, \end{aligned}$$

which gives that $n_{s}-1=-2n_{0}.~$Recall that inflation ends at $\omega _{f},$ where $\varepsilon _{H}\left( \omega _{f}\right) =1$. Hence, we find that

38 $$\begin{aligned} \omega _{f}=\frac{3}{n_{0}}\ln \left[ \frac{F_{0}}{F_{1}\left( n_{0}-1\right) }\right] , \end{aligned}$$

and

39 $$\begin{aligned} n_{s}\left( n_{0},N_{e}\right) -1=-2n_{0},\quad r\left( n_{0},N_{e}\right) =\frac{10n_{0}}{1+\left( n_{0}-1\right) e^{-2n_{0}N_{e}}}, \end{aligned}$$

where $N_{e}$ is the number of e-folds; recall that $6N_{e}=\omega _{f} -\omega _{i}.$

**Fig. 1:**
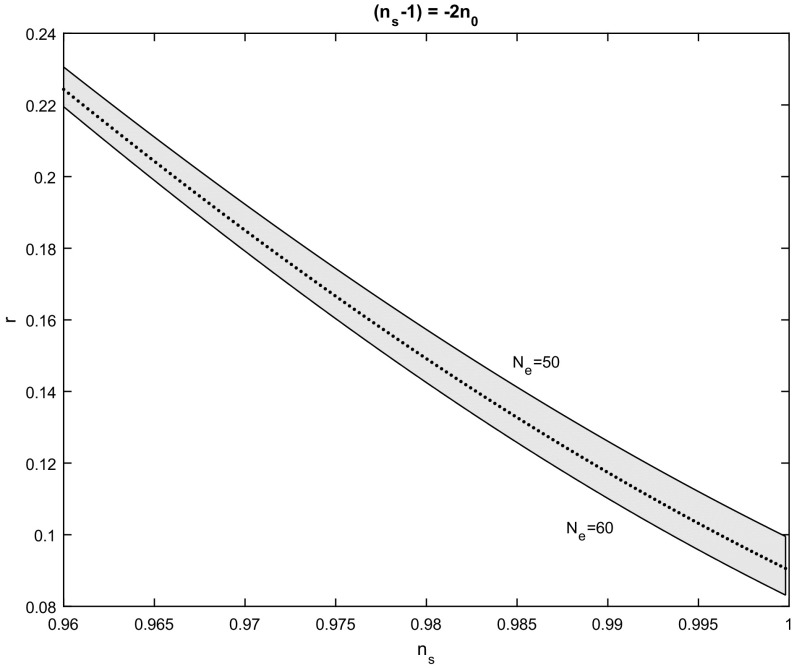
Spectral index $n_{s}$ to scalar to tensor ratio *r*,  for the scalar field potential in which $n_{s}-1=-2n_{0}.~$The figure is for various values of $n_{0}$ in the range $n_{0}\in \left( 0,0.02\right)$ and for number of e-folds $N_{e}\in \left[ 50,60\right]$. The dot line is for $N_{e}=55$

From the latter expressions, it follows that while the value of $n_{0}$ fixes the index $n_{s}$, only the scalar-tensor ratio *r* depends on the number of e-folds. Furthermore, the integration constants are non-essential and fix the value of the scale factor at the end of the inflation. In Fig. 1 the $n_{s}-r$ plot is presented for the expressions (39) and for $n_{0}\in \left( 0,0.02\right)$, $\ N_{e}\in \left[ 50,60\right]$. Note that for values of $n_{0}$ where $n_{s}$ is constrained by the Planck 2015 data, it follows that $r<0.11$ for very large values of $N_{e}$, while for the number of e-folds that we considered in the figure $r>0.11$.

Furthermore, in the case of the Harrison–Zeldovich spectrum, that is, $n_{0}=0$, we calculate $r=\frac{10}{1-2N_{e}}$, hence $r<0.11$ when $N_{e}>50$. As before, the integration constants (now $F_{1}$ and $\omega _{0}$) specify only the value of $\omega _{f}$ at which inflation ends. We calculate that $\omega _{f}=3+\omega _{0}$.

### Linear expression: $n_{s}-1\simeq r$

We continue now by taking the more general ansatz, $n_{s}-1=-2n_{1} \varepsilon _{H}-2n_{0}$; that is, the spectral index $n_{s}$ depends linearly on the tensor to scalar ratio, *r*. Recall that $r=10\varepsilon _{H}$; so in the limit in which $n_{1}\rightarrow 0$ we are in a situation where $n_{s}-1=~const$. We study two cases: $n_{0}=0$ and $n_{0}\ne 0$.

#### Zero $n_{0}$:

When $n_{0}=0$, so $n_{s}=1$ and $r=0$, with the use of (18) the differential equation for $F(\omega )$ is

40 $$\begin{aligned} F^{\prime \prime }+\left( 1-n_{1}\right) \left( F^{\prime }\right) ^{2}=0, \end{aligned}$$

with solution

41 $$\begin{aligned} F\left( \omega \right) =-\frac{1}{n_{1}-1}\ln \left( F_{1}\left( \omega -\omega _{0}\right) \right) ,\quad n_{1}\ne 1, \end{aligned}$$

or

42 $$\begin{aligned} F\left( \omega \right) =F_{1}\left( \omega -\omega _{0}\right) ,\quad n_{1}=1, \end{aligned}$$

where $F_{1}$ is constant. Of course, one should be careful because we have assumed that $n_{s}$ is given in terms of the first approximation, i.e., $\left( \varepsilon _{H}\right) ^{2}\simeq 0,$ and second-order approximations may need to be considered. For simplicity, we continue with the first-order approximations. The ansatz is stronger if $n_{1}$ is of order $O\left( \varepsilon _{H}\right) ^{-1}$.

Obviously, for $n_{1}=0$, eq. (26) is recovered. Equation (41) corresponds to the solution of the generalized Chaplygin gas, (28), with $\lambda =2-n_{1}.~$ The scalar-field potential is given by the expression [56]

43 $$\begin{aligned} V\left( \phi \right) \varpropto \phi ^{-\frac{2}{1-\lambda }}\left( 1-\frac{2}{3\left( 1-\lambda ^{2}\right) }\phi ^{-2}\right) \end{aligned}$$

and the scale factor is that of intermediate inflation $a\left( t\right) \simeq \exp \left( a_{1}t^{N}\right) ~$for$~n\ne \frac{3}{2}~$and $a\left( t\right) \simeq \exp \left( a_{1}e^{{\bar{\gamma }}t}\right)$ for $~n=\frac{3}{2}$. Moreover, the effective EoS is derived to be

44 $$\begin{aligned} w_{\phi }\left( \omega \right) =-1+\frac{2}{\left( 1-n_{1}\right) }\left( \omega -\omega _{0}\right) ^{-1}. \end{aligned}$$

In the limit where $n_{1}=1,$ from solution (42) we calculate $w_{\phi }\left( \omega \right) =-1+2F_{1}$, which is a particular solution of the exponential potential $V\left( \phi \right) =\frac{\left( 1-F_{1} \right) }{12}e^{-\sqrt{6F_{1}}\phi }$, and there is a power-law scale factor $a\left( t\right) \varpropto t^{\frac{1}{3F_{1}}}$.

#### Non-zero $n_{0}$:

Now we assume that $n_{0}\ne 0$. The unknown function, $F\left( \omega \right)$, which provides the solution for the spacetime metric, satisfies the second-order nonlinear differential equation

45 $$\begin{aligned} F^{\prime \prime }+\left( 1-n_{1}\right) \left( F^{\prime }\right) ^{2} -\frac{n_{0}}{3}F^{\prime }=0 \end{aligned}$$

with closed-form solution

46 $$\begin{aligned} F\left( \omega \right) =-\frac{1}{n_{1}-1}\ln \left( F_{1}\exp \left( \frac{n_{0}}{3}\omega \right) +F_{0}\right) ,\quad n_{1}\ne 1, \end{aligned}$$

or

47 $$\begin{aligned} F\left( \omega \right) =F_{1}\exp \left( \frac{n_{0}}{3}\omega \right) +F_{0},\quad n_{1}=1. \end{aligned}$$

As in the case of $n_{0}=0$, when $n_{1}\ne 1$ the solution generalizes that of (32) and the scalar field now satisfies the equation of state of the generalized Chaplygin gas, namely

48 $$\begin{aligned} p_{\phi }=A\rho _{\phi }^{\lambda }+B\rho _{\phi }, \end{aligned}$$

where, in contrast to (33) where $\lambda =2$, we now have $\lambda =2-n_{1}$.

Again, the scalar-field potential is given in terms of the hyperbolic functions as [52]

49 $$\begin{aligned} V\left( \phi \right) =\frac{1}{36}\left( \frac{e^{-\sqrt{\frac{n_{0}\left( \lambda -1\right) }{3}}\phi }}{4F_{1}}\left( 1+e^{\sqrt{\frac{n_{0}\left( \lambda -1\right) }{3}}\phi }\right) ^{2}\right) ^{\frac{1}{1-\lambda } }\left( 3-\frac{n_{0}}{\lambda -1}\left( \frac{e^{\sqrt{\frac{n_{0}\left( \lambda -1\right) }{3}}\phi }-F_{0}F_{1}}{e^{\sqrt{\frac{n_{0}\left( \lambda -1\right) }{3}}\phi }+F_{0}F_{1}}\right) ^{2}\right) . \nonumber \\ \end{aligned}$$

Alternatively, for $n_{1}=1,$ the scalar-field potential is

50 $$\begin{aligned} V\left( \phi \right) =\frac{1}{72}\exp \left( -\frac{n_{0}}{2}\phi ^{2} -F_{0}\right) \left( 6-\left( n_{0}\phi \right) ^{2}\right) . \end{aligned}$$

The scale factor, $a\left( t\right)$, cannot be written as a closed-form expression in either case. However, for the effective EoS parameter we have

51 $$\begin{aligned} w_{\phi }\left( \omega \right) =-1+\frac{2}{3}n_{0}F_{1}\exp \left( \frac{n_{0}}{3}\omega \right) ,\quad n_{1}=1 \end{aligned}$$

and

52 $$\begin{aligned} w_{\phi }=-1+\frac{2}{3}\frac{n_{0}F_{1}\exp \left( \frac{n_{0}}{3} \omega \right) }{1-n_{1}}\left( F_{1}\exp \left( \frac{n_{0}}{3} \omega \right) +F_{0}\right) ^{-1},\quad n_{1}\ne 1. \end{aligned}$$

So far, the generalized Chaplygin gas which leads to intermediate inflation, and another generalization of the Chaplygin gas which was studied in [52], have been recovered. For these two inflationary models the scalar-field potentials have similar forms. For one model the potential, $V\left( \phi \right)$, is given by a polynomial function of $\phi$, while for the second model it is given as a function of the hyperbolic trigonometric functions.

#### Observational constraints

The slow-roll parameters for the solution (41) are calculated to be

53 $$\begin{aligned} \varepsilon _{H}=\frac{3}{1-n_{1}}\left( \omega -\omega _{0}\right) ^{-1},\quad \eta _{H}=\left( n_{1}-2\right) \varepsilon _{H}. \end{aligned}$$

Hence, $\omega _{f}=\frac{3}{1-n_{1}}+\omega _{0},$ from which we find

54 $$\begin{aligned} n_{s}\left( n_{1},N_{e}\right) =1+\frac{2n_{1}}{2\left( n_{1}-1\right) N_{e}-1},\quad r\left( n_{1},N_{e}\right) =\frac{10}{1-2\left( n_{1}-1\right) N_{e}}. \end{aligned}$$

**Fig. 2:**
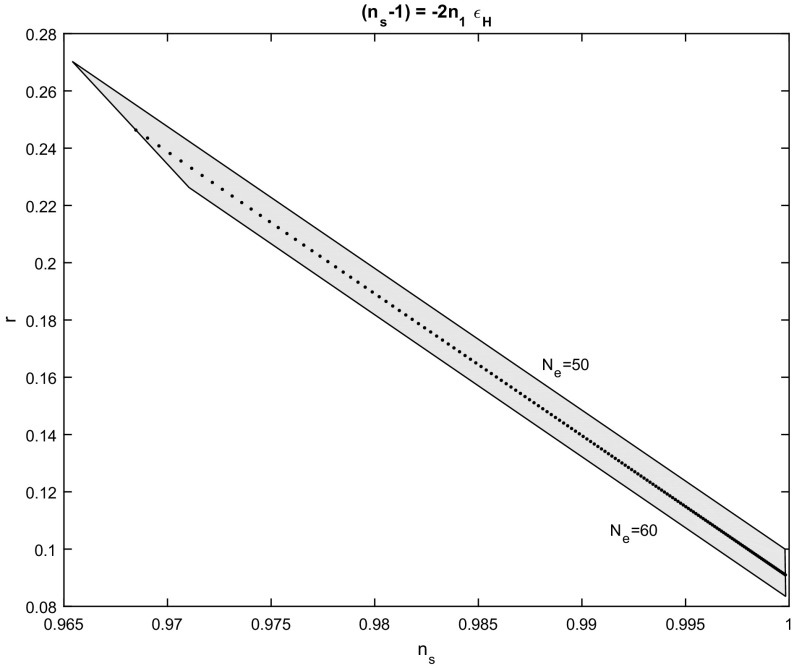
Spectral index $n_{s}$ to scalar to tensor ratio *r*,  for the scalar field potential in which $n_{s}-1=-2n_{1}\varepsilon _{H}.~$The figure is for various values of $n_{1}$ in the range $n_{1}\in \left( 0.01,0.65\right)$ and for number of e-folds $N_{e}\in \left[ 50,60\right]$. The dot line is for $N_{e}=55$

As before, the constants of integration have no effect on the inflationary parameters. Furthermore, we see that we have $n_{s}\left( N_{e}\right) -1<0$, so necessarily $n_{1}>0$ and $n_{1}<1+\frac{1}{2N_{e}}$, while the latter also ensures that $r\left( N_{e}\right) >0.$ The case in which $n_{1}=1$ corresponds to the exponential potential and and gives constant slow-roll parameters. The $n_{s}-r$ diagram for the expressions (54) is given in Fig. 2, for $N_{e}\in \left[ 50,60\right]$ and the free parameter $n_{1},$ with $n_{1}\in \left( 0.01,0.65\right)$.

For $n_{0}\ne 0$, from (46) it follows that

55 $$\begin{aligned} \varepsilon _{H}{=}\frac{n_{0}}{n_{1}-1}\left( 1{+}\frac{F_{0}}{F_{1}} e^{-\frac{n_{0}}{3}\omega }\right) ~^{-1},\quad \ \eta _{H}{=}\frac{n_{0}}{n_{1} {-}1}\left( \frac{F_{1}e^{\frac{n_{0}}{3}\omega }{+}F_{0}\left( n_{1}-1\right) }{F_{1}e^{\frac{n_{0}}{3}\omega }+F_{0}}\right) , \end{aligned}$$

which shows that inflation ends when

56 $$\begin{aligned} \omega _{f}=\frac{3}{n_{0}}\ln \left( \frac{F_{0}}{F_{1}}\frac{\left( 1-n_{1}\right) }{n_{0}-\left( 1-n_{1}\right) }\right) , \end{aligned}$$

from which we find

57 $$\begin{aligned} n_{s}\left( n_{0},n_{1},N_{e}\right)= & {} 1-\frac{2n_{0}\left( 1+\left( n_{0}+n_{1}-1\right) e^{-2n_{0}N_{e}}\right) }{\left( 1-n_{1}\right) +\left( n_{0}+n_{1}-1\right) e^{-2n_{0}N_{e}}}~, \end{aligned}$$

58 $$\begin{aligned} ~~r\left( n_{0},n_{1},N_{e}\right)= & {} \frac{10n_{0}}{\left( 1-n_{1}\right) +\left( n_{0}+n_{1}-1\right) e^{-2n_{0}N_{e}}}. \end{aligned}$$

**Fig. 3:**
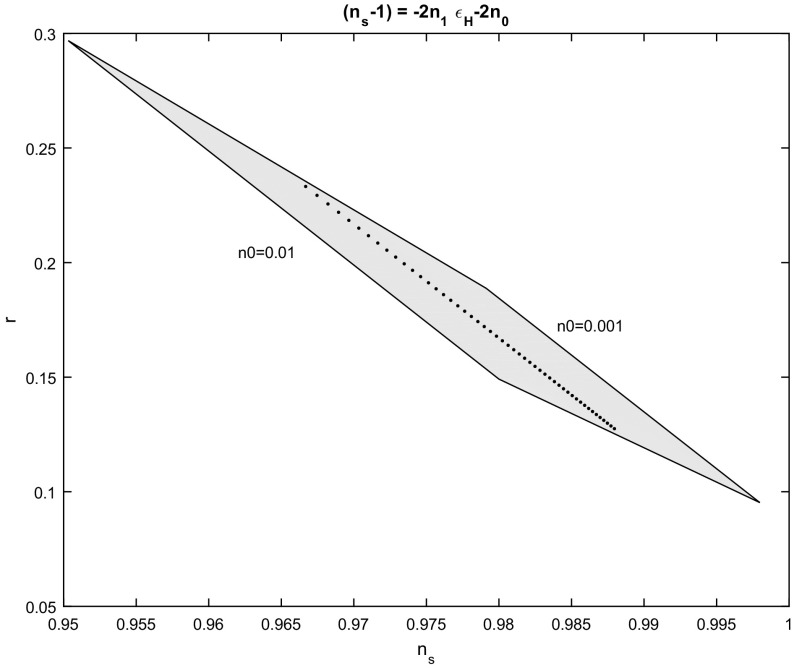
Spectral index $n_{s}$ to scalar to tensor ratio *r*,  for the scalar field potential in which $n_{s}-1=-2n_{1}\varepsilon _{H}-2n_{0}.~$The figure is for various values of the parameters $n_{0}$ and $n_{1};$ in the range $n_{0}\in \left[ 0.001,0.01\right]$, $n_{1}\in \left[ 0.001,0.5\right]$ and for number of e-folds $N_{e}=55$. The dot line is for $n_{0}=0.005$

**Fig. 4:**
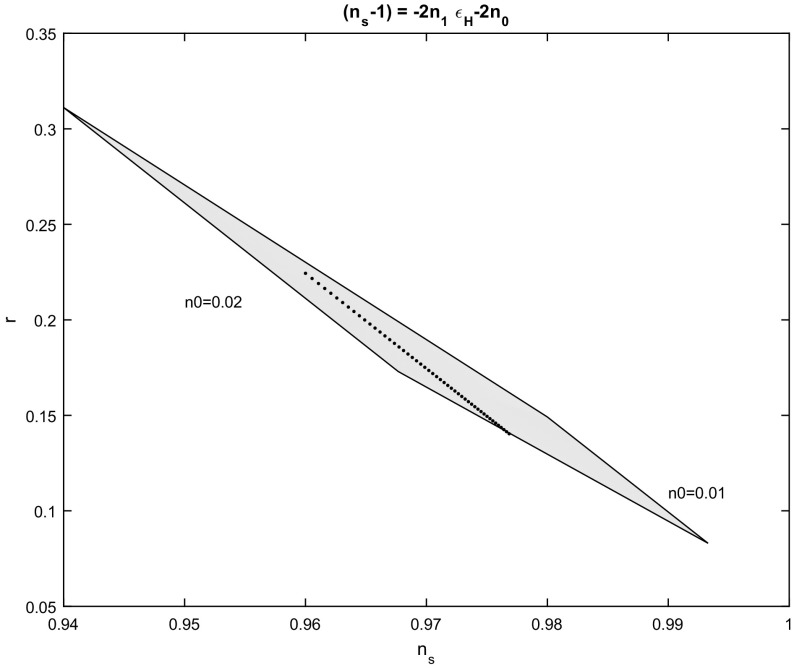
Spectral index $n_{s}$ to scalar to tensor ratio *r*,  for the scalar field potential in which $n_{s}-1=-2n_{1}\varepsilon _{H}-2n_{0}.~$The figure is for various values of the parameters $n_{0}$ and $n_{1};$ in the range $n_{0}\in \left[ 0.01,0.03\right]$, $n_{1}\in \left[ -\,0.5,0.001\right]$ and for number of e-folds $N_{e}=55$. The dot line is for $n_{0}=0.02$

The $n_{s}-r$ diagram for the parameters (57), (58) is given in Figs. 3 and 4, for the number of e-folds $N_{e}=55$ and for various values of the free parameters $n_{0}$ and $n_{1}$. Figure 3 is for $n_{0}\in \left[ 0.001,0.01\right]$ and $n_{1}\in \left[ 0.001,0.5\right]$, while Fig. 4 is for $n_{0}\in \left[ 0.01,0.03\right]$ and $n_{1}\in \left[ -\,0.5,-\,0.001\right]$.

We continue our analysis with a more general case in which the relation between $n_{s}$ and *r* is parabolic.

### Parabolic: $n_{s}-1\simeq r^{2}$

Consider now the case where the relation $n_{s}-1=h\left( \varepsilon _{H}\right)$ describes a parabola such that

59 $$\begin{aligned} n_{s}-1=2n_{2}\left( \varepsilon _{H}\right) ^{2}-2n_{1}\varepsilon _{H}-2n_{0}. \end{aligned}$$

From the constraint equation above, the nonlinear differential equation for $F\left( \omega \right)$ is

60 $$\begin{aligned} F^{\prime \prime }+3n_{2}\left( F^{\prime }\right) ^{3}+\left( 1-n_{1}\right) \left( F^{\prime }\right) ^{2}-\frac{n_{0}}{3}F^{\prime }=0, \end{aligned}$$

which can be written as a first-order ordinary differential equation in terms of the effective EoS parameter or in terms of the HSR parameter, $\varepsilon _{H}$, as

61 $$\begin{aligned} 3\varepsilon _{H}^{\prime }+n_{2}\left( \varepsilon _{H}\right) ^{3}+\left( 1-n_{1}\right) \left( \varepsilon _{H}\right) ^{2}-n_{0}\varepsilon _{H}=0. \end{aligned}$$

As in the linear case, for completeness, one has to consider the second-order approximation in the definition of $n_{s}\left( \varepsilon _{H},\eta _{H}\right)$. However, we continue just with the first approximation here. The ansatz is consistent if $n_{2}~$is of the order $n_{2}\simeq \left( \varepsilon _{H}\right) ^{-2}.$

The general solution of eqn. (61) is

62 $$\begin{aligned}&\frac{3}{2n_{0}}\ln \left( n_{2}+\frac{\left( 1-n_{1}\right) }{\varepsilon _{H}}-\frac{n_{0}}{\left( \varepsilon _{H}\right) ^{2}}\right) \nonumber \\&\quad -\frac{3\left( 1-n_{1}\right) \arctan \left( \frac{2n_{2}\varepsilon _{H}+\left( 1-n_{1}\right) }{\sqrt{4n_{0}n_{2}+\left( 1-n_{1}\right) ^{2} }}\right) }{n_{0}\sqrt{4n_{0}n_{2}+\left( 1-n_{1}\right) ^{2}}}=\left( \omega -\omega _{0}\right) , \end{aligned}$$

which for some specific values of the free parameters can be written in closed form.

In the special case of $n_{1}=1$ we find that

63 $$\begin{aligned} \left( \varepsilon _{H}\right) ^{2}=\frac{n_{0}}{c_{1}e^{-\frac{2}{3} n_{0}\omega }+n_{2}},\quad n_{0}\ne 0, \end{aligned}$$

and

64 $$\begin{aligned} \left( \varepsilon _{H}\right) ^{2}=\frac{3}{2n_{2}\omega +c_{1}},\quad n_{0}=0. \end{aligned}$$

Hence, for the function $F\left( \omega \right)$ defining the metric, we have

65 $$\begin{aligned} F\left( \omega \right) =\pm \frac{{\text {arctanh}}\left( \sqrt{1+\frac{c_{1}}{n_{2}}}e^{-\frac{2}{3}n_{0}\omega }\right) }{\sqrt{n_{0}n_{2} }},\quad n_{0}\ne 0, \end{aligned}$$

and

66 $$\begin{aligned} F\left( \omega \right) =\pm \sqrt{3}\sqrt{\frac{2}{n_{2}}\omega +c_{1}},\quad n_{0}=0. \end{aligned}$$

Therefore, from (65), the potential is found to be

67 $$\begin{aligned} V\left( \phi \right) \varpropto \frac{1}{12}\exp \left( -V_{1}\phi ^{2/3}\right) \left( 1+V_{2}\phi ^{-\frac{2}{3}}\right) , \end{aligned}$$

where $V_{1,2}=V_{1,2}\left( n_{2}\right)$ are constant, and it can be seen that it has the form of the potential in (50). For small values of $\left| \phi \right| ,$the potential, (67) becomes the power-law potential $V\left( \phi \right) \simeq \phi ^{-\frac{2}{3}}$, which means that finite-time singularities of the ’generalized sudden’ type can follow [62]. Moreover, for the EoS for the scalar field it follows that the effective equation of state is

68 $$\begin{aligned} p_{\phi }=\left( \frac{6\rho _{\phi }}{n_{2}\ln \left( 12\rho _{\phi }\right) }-\rho _{\phi }\right) ,\quad n_{0}=0. \end{aligned}$$

On the other hand, for $n_{0}\ne 0$ we find that that the EoS is

69 $$\begin{aligned} p_{\phi }=2\sqrt{\frac{n_{0}}{n_{2}}}\left( \frac{16n_{0}n_{2}\rho _{\phi } ^{2}+1}{16n_{0}n_{2}\rho _{\phi }^{2}-1}\right) -\rho _{\phi },\quad n_{0}\ne 0. \end{aligned}$$

The scalar-field potential for $n_{0}\ne 0$ cannot be written in closed form. However, in terms of $\omega$ it is

70 $$\begin{aligned} V\left( \omega \right) =e^{-F\left( \omega \right) }\left( 1\pm \sqrt{\frac{n_{0}}{n_{2}}\left( 1+\frac{c_{1}}{n_{2}}e^{-\frac{2}{3}n_{0}\omega }\right) ^{-1}}\right) . \end{aligned}$$

#### Observational constraints

For the solution (66), in which $n_{0}=0$ and $n_{1}=1$, we find that inflation ends when $\omega _{f}=\frac{27-c_{1}\left( n_{2}\right) ^{2} }{2n_{2}}$, and the parameters $n_{s}$ and *r* are given in terms of the number of e-folds by

71 $$\begin{aligned} n_{s}-1=\frac{2n_{2}-6\sqrt{9+4n_{2}N_{e}}}{9+4n_{2}N_{e}},\quad r=\frac{30}{\sqrt{9+4n_{2}N_{e}}}. \end{aligned}$$

**Fig. 5:**
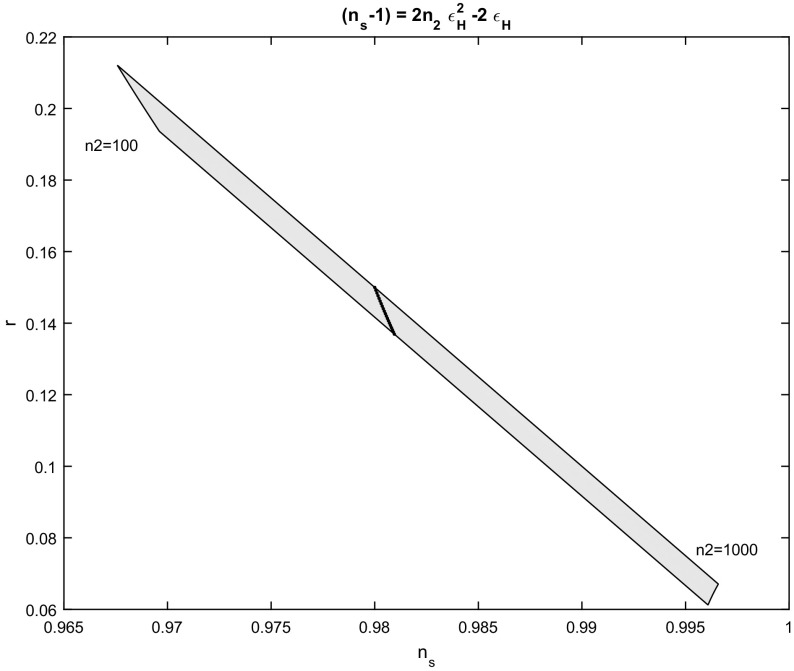
Spectral index $n_{s}$ to scalar to tensor ratio *r*,  for the scalar field potential in which $n_{s}-1=2n_{2}\left( \varepsilon _{H}\right) ^{2}-2\varepsilon _{H}.~$The figure is for various values of the parameter $n_{2}\in \left( 10^{2},10^{3}\right)$ and number of e-folds $N_{e} \in \left[ 50,60\right]$. The dot line is for $n_{2}=2\times 10^{2}$

In Fig. 5 the $n_{s}-r$ diagram is given for the parameters (71) with $n_{2}\in \left( 10^{2},10^{3}\right)$ and $N_{e} \in \left[ 50,60\right]$. Note that in order for this case to differ from the linear we have assumed that $n_{2}$ has a large value of order $\left( \varepsilon _{H}\right) ^{-1}$.

**Fig. 6:**
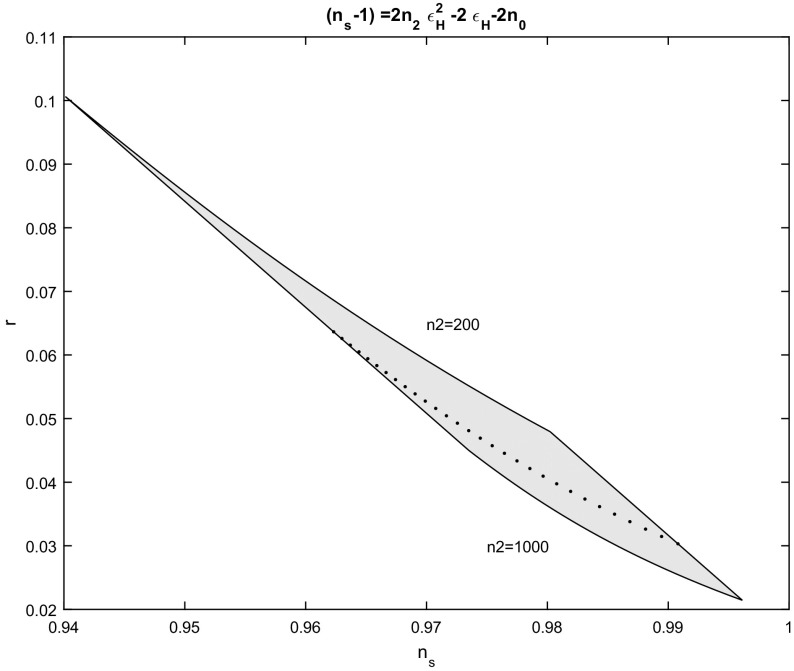
Spectral index $n_{s}$ to scalar to tensor ratio *r*,  for the scalar field potential in which $n_{s}-1=2n_{2}\left( \varepsilon _{H}\right) ^{2}-2\varepsilon _{H}-2n_{0}.~$The figure is for various values of the parameter $n_{2}\in \left[ 2\times 10^{2},10^{3}\right]$ and $n_{0}\in \left[ 10^{-4},0.3\right]$ while for the number of e-folds we sellected $N_{e}=55$. The dot line is for $n_{2}=5\times 10^{2}$

Similarly, for the solution in which $n_{0}\ne 0$ but $n_{1}=1$; that is, for the expression (65), we omit the derivation of the parameters $n_{s}-r$. However, in Fig. 6 the $n_{s}-r$ diagram is presented for a number of e-folds given by $N_{e}=55$ and $n_{0}\in \left[ 10^{-4} ,0.3\right]$ and $n_{2}=\left[ 2\times 10^{2},10^{3}\right]$.

## Conditions to escape from Inflation

It is an open question as to which values for the free parameters of our models determine when inflation ends. In order to answer this, we consider the master equation (60) and specifically we choose to rewrite it in terms of the HSR parameter $\varepsilon _{H}\left( \omega \right)$ as,

72 $$\begin{aligned} 3\varepsilon _{H}^{\prime }=\left( n_{0}+\left( n_{1}-1\right) \varepsilon _{H}-n_{2}\varepsilon _{H}^{2}\right) \varepsilon _{H}. \end{aligned}$$

This equation has the following critical points:

73 $$\begin{aligned} \varepsilon _{H}^{\left( 0\right) }=0,\quad \varepsilon _{H}^{\left( \pm \right) }=\frac{n_{1}-1\pm \sqrt{\left( 1-n_{1}\right) ^{2}+4n_{0}n_{2}}}{2n_{2} },\quad \text {for}~n_{2}\ne 0, \end{aligned}$$

or

74 $$\begin{aligned} \varepsilon _{H}^{\left( 0\right) }=0,\quad \varepsilon _{H}^{\left( 1\right) }=\frac{n_{0}}{1-n_{1}},\quad \text {when}~n_{2}=0~\text {and }n_{1}\ne 1. \end{aligned}$$

Hence, in order for inflation to end in the cosmological models that we studied, the free parameters of the models have to be constrained so that one of the critical points, $\varepsilon _{H}^{\left( \pm \right) }$ or $\varepsilon _{H}^{\left( 1\right) }$, is an attractor, and also that $\varepsilon _{H}^{\left( \pm \right) }\ge 1$ or $\varepsilon _{H}^{\left( 1\right) }\ge 1$. We note that point $\varepsilon _{H}^{\left( 0\right) }$ describes a de Sitter universe (that is, $w_{\phi }=-1$), while for the other critical points the equation of state parameter, $w_{\phi }$, is constant. Therefore, from the previous analysis we see that at the critical points the scalar field potential is described by the exponential function. 

We proceed by considering the cases (a) $n_{2}=0$ and (b)  $n_{2}\ne 0$, where the number of critical points differs.

### Subcase $n_{2}=0$

Let as assume the simple case which corresponds to the master equation (45); that is, $n_{2}=0~$ and we assume that $n_{1}\ne 1$. In that consideration, the critical points of the system are the $\varepsilon _{H}^{\left( 0\right) }~$ and $\varepsilon _{H}^{\left( 1\right) }~$of (74).

As far as concerns the stability of these points, we find that point $\varepsilon _{H}^{\left( 1\right) }$ is the unique attractor of the equation when $n_{0}>0$, and $\varepsilon _{H}^{\left( 1\right) }$ describes a point without acceleration when $n_{1}<1$ and $n_{0}>1-n_{1}$. On the other hand, when $n_{0}<0$, the unique attractor of the system is the de Sitter point $\varepsilon _{H}^{\left( 0\right) },$ although in this case the model does not provide an exit from inflation.

### Subcase $n_{2}\ne 0$

For $n_{2}\ne 0,$ a necessary condition for an exit from the inflation to occur, is that the critical points $\varepsilon _{H}^{\left( \pm \right) }$ are real; that is, $4n_{0}n_{2}\ge -\frac{\left( 1-n_{1}\right) ^{2}}{4}$. In the special limit in which $n_{0}=0$, the points $\varepsilon _{H}^{\left( \pm \right) }$ reduce to $\varepsilon _{H}^{\left( 0\right) }$ and $\varepsilon _{H}^{\left( 2\right) }=\frac{n_{1}-1}{n_{2}}$. In that case, the two points are stable when $n_{2}>0$, and $\varepsilon _{H}^{\left( 2\right) }$ is positive for any value of $n_{1}>1$.

In the general scenario with $n_{0}\ne 0,$ it follows easily that in order for $\varepsilon _{H}^{\left( 0\right) }$ to be an elliptic point we require $n_{0}>0$. Moreover, by assuming the condition $\varepsilon _{H}^{\left( \pm \right) }>1,$ we find that only the point $\varepsilon _{H}^{\left( +\right) }$ can be an attractor outside the inflationary era and this is possible only when the free parameters satisfy the conditions

75 $$\begin{aligned} (i)~n_{2}<0,~n_{1}<1+2n_{2},~n_{0}>1-n_{1}+n_{2}\text { and }4n_{0} n_{2}\ge -\frac{\left( 1-n_{1}\right) ^{2}}{4}, \end{aligned}$$

or

76 $$\begin{aligned} (ii)~n_{2}>0,~n_{1}>1+n_{2}~\text { and }n_{0}>1-n_{1}+n_{2}, \end{aligned}$$

or

77 $$\begin{aligned} (iii)~n_{2}>0, \quad n_{1}\le 1+n_{2}~\text { and }n_{0}>0. \end{aligned}$$

Hence, for values of the free parameters in those ranges only the third model, i.e. where $h\left( r\right)$ is a quadratic function, admits an attractor outside the inflationary era.

**Fig. 7:**
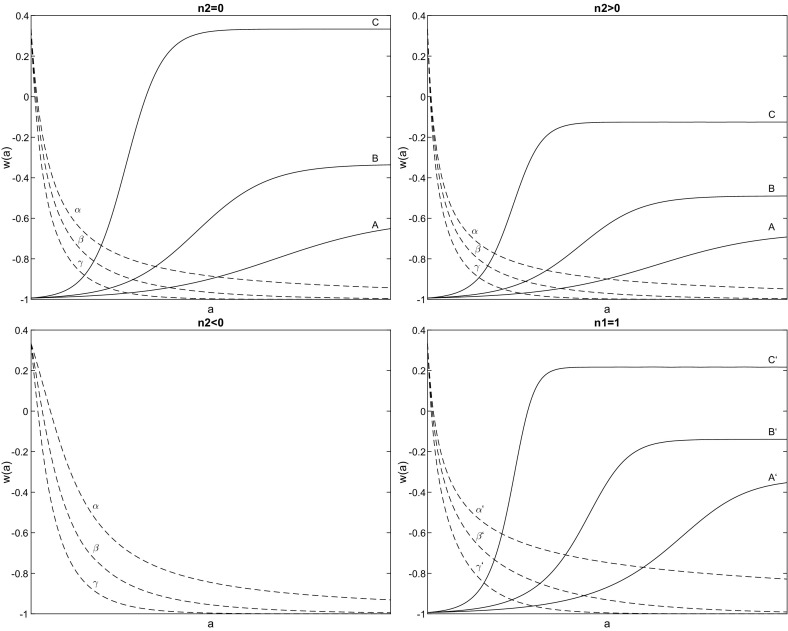
Qualitative evolution of the equation of state parameter $w\left( a\right)$, given by the solution of eq. (72). The solid lines are for initial condition $\varepsilon _{H}\left( a_{0}\right) =0.01$ while the dash-dash lines are for initial conditions $\varepsilon _{H}\left( a_{0}\right) =2$. As far as concerns the parameters ${\mathbf {n}}\left( X\right) =\left( n_{0},n_{1}\right)$ we have that ${\mathbf {n}}\left( A\right) =\left( 0.3,0.5\right) ,~{\mathbf {n}}\left( B\right) =\left( 0.5,0.5\right)$, ${\mathbf {n}}\left( C\right) =\left( 1,0.5\right) ,~{\mathbf {n}}\left( \alpha \right) =\left( -0.1,0.5\right)$, ${\mathbf {n}} \left( \beta \right) =\left( -0.2,0.5\right)$ and ${\mathbf {n}}\left( c\right) =\left( -0.5,0.5\right)$. Upper-left fig. is for $n_{2}=0$, upper-right fig. is for $n_{2}=0.2$, and lower-left fig. is for $n_{2} =-0.2.~$Furthermore, the free parameters on the lower-right figure are ${\mathbf {n}}^{\prime }\left( X\right) =\left( n_{0},n_{2}\right) ,$ such that ${\mathbf {n}}\left( A\right) =\left( 0.3,0.3\right) ,~{\mathbf {n}}\left( B\right) =\left( 0.5,0.3\right)$, ${\mathbf {n}}\left( C\right) =\left( 1,0.3\right) ,~{\mathbf {n}}\left( \alpha \right) =\left( -0.1,0.3\right)$, ${\mathbf {n}}\left( \beta \right) =\left( -0.2,0.3\right)$ and ${\mathbf {n}}\left( c\right) =\left( -0.5,0.3\right)$ while $n_{1}=1$. The values of the free parameters have been chosen such that to cover the stability analysis of eq. (72)

In Fig. 7 the qualitative evolution of the equation of state parameter $w\left( a\right)$, given by the solution of eq. (72) is presented for various values of the free parameters.

## Equivalent transformations

It is interesting that, when we set $n_{s}-1=0$, the scalar field mimics the generalized Chaplygin gas (28) with $\lambda =2$. Yet, when we assumed that $\lambda \ne 2$ in the equation of state of the generalized Chaplygin gas, we found that $n_{s}-1=-2n_{1}\varepsilon _{H}$, where $\lambda =2-n_{1}$. These two models are the solutions of the two different master equations, (26) and (40), respectively. Yet, these two equations are different for $n_{1}\ne 0$, we observe that there exists a transformation $F\left( \omega \right) \rightarrow {\bar{F}}\left( \omega \right)$, allowing eq. (26) to be written in the form of (40) and vice versa.

Suppose that $n_{1}\ne 0,1$, then if in (26) we substitute

78 $$\begin{aligned} F\left( \omega \right) \rightarrow \left( 1-n_{1}\right) {\bar{F}}\left( \omega \right) , \end{aligned}$$

equation (26) becomes

79 $$\begin{aligned} {\bar{F}}^{\prime \prime }+\left( 1-n_{1}\right) \left( {\bar{F}}^{\prime }\right) =0 \end{aligned}$$

which is just eq. (31). The transformation alters the line element of the FLRW spacetime (15) to

80 $$\begin{aligned} ds^{2}=-\left( e^{-{\bar{F}}\left( \omega \right) }\right) ^{\left( 1-n_{1}\right) }d\omega ^{2}+e^{\omega /3}(dx^{2}+dy^{2}+dz^{2}). \end{aligned}$$

A similar observation holds for the master equations (31) and (45). Under the transformation, (78), these two equations are related, so a known solution for the model with EoS (48) for a specific $\lambda$ can be used to construct a solution for another cosmological model with a similar EoS parameter but with some other constant $\lambda$. For completeness, note that in the case of $n_{1}=1$, the transformation which relates the different set of equations is not that of (78) but $F\left( \omega \right) =\ln \left( {\bar{F}}\left( \omega \right) \right)$.

On the other hand, it is important to mention that eq. (40) can be written in the form of (26) under the simple change of variable $\omega =\frac{3}{n_{0}}\ln \left( {\bar{\omega }}\right)$. The same transformation can be applied in the master equation, (45), which is transformed into equation (40). Moreover, if we also apply the transformation (78) to (45), then the latter takes the form of the master equation, (26).

These two transformations modify the line element of the FLRW spacetime (15) to

81 $$\begin{aligned} d{\bar{s}}^{2}=-\frac{9}{\left( n_{0}\right) ^{2}\omega ^{2}}\left( e^{-{\bar{F}}\left( {\bar{\omega }}\right) }\right) ^{\left( 1-n_{1}\right) }d{\bar{\omega }}^{2}+\left( {\bar{\omega }}\right) ^{1/n_{0}}(dx^{2} +dy^{2}+dz^{2}). \end{aligned}$$

Moreover, in the limit for which $n_{1}=1$ in (40) the latter becomes the equation of a free particle, while the resulting scalar field theory is that of the exponential potential where the scalar field has a constant EoS parameter.

The existence of transformations of this kind, which transform the one model into another, is not a coincidence. The master equations (26), (31), (40) and (45) are maximally symmetric. In particular they are invariant under the action of one-parameter point transformations (Lie point symmetries) which form the $SL\left( 3,R\right)$ Lie algebra.^2^

Consider now the classical Newtonian analogue of a free particle and an observer whose measuring instruments for time and distance are not linear. By using the measured data of the observer we reach in the conclusion that it is not a free particle. On the other hand, in the classical system of the harmonic oscillator an observer with nonlinear measuring instruments can conclude that the system observed is that of a free particle, or that of the damped oscillator or another system. From the different observations, various models can be constructed. However, all these different models describe the same classical system and the master equations are invariant under the same group of point transformations but in different parametrization.

In the master equations that we studied there is neither position nor time variables: the independent variable is the scale factor $\omega =6\ln a$, and the Hubble function is the dependent variable, $H\left( a\right)$. Therefore, we can say that at the level of the first-order approximation for the spectral indices, various representations of the variables $\left\{ a,H\left( a\right) \right\}$ provide different observable values for the spectral indices. This property is violated when we consider the second-order approximation.

Transformations of this kind are well-known in physics. For instance, the Darboux transformation for the Schrödinger equation [60] is just a point transformation that relates linear equations with maximal symmetry; that is, it belongs to exactly the same category of transformations that we discuss here. A special characteristic of the Darboux transformation is that it preserves the form of the equation but the potential in the Schrödinger equation changes. An application of the Darboux transformation for the determination of exactly solvable cosmological models can be found in [61].

Transformations which keep the form of our master equation exist. We do not have potential terms in the master equations but there are transformations which change the constant coefficients appearing there while retaining the form of the master equations.

In order to demonstrate this, consider the master equation (45). The application of the first transformation $F\left( \omega \right) \rightarrow \frac{1-{\bar{n}}_{1}}{1-n_{1}}{\bar{F}}\left( \omega \right) ~$in (45), preserves the form of the master equation but the constant $\lambda$ in the equation of state for the generalized Chaplygin gas (48) shifts from $\lambda =2-n_{1}$ to ${\bar{\lambda }}=2-{\bar{n}}_{1}$. Moreover, the application of the second transformation, $\omega \rightarrow \frac{{\bar{n}}_{0}}{n_{0}}{\bar{\omega }},$ in (45) gives

82 $$\begin{aligned} \frac{d^{2}{\bar{F}}}{d{\bar{\omega }}^{2}}+\left( 1-{\bar{n}}_{1}\right) \left( \frac{d{\bar{F}}}{d{\bar{\omega }}}\right) ^{2}-\frac{{\bar{n}}_{0}}{3}\frac{d{\bar{F}}}{d{\bar{\omega }}}=0 \end{aligned}$$

which is exactly the same master equation, just with different coefficients.

Furthermore, for the more general case that we studied (the master equation of eq. (60)) it is easy to see that for $n_{2}n_{0}\ne 0$, eq. (60) admits eight Lie point symmetries; that is, it is maximally symmetric. Hence, there exists a mapping $\left\{ \omega ,F\left( \omega \right) \right\} \rightarrow \left\{ \Omega ,\Phi \left( \Omega \right) \right\}$ which transforms the master equation (60) to that of a free particle, or to any other maximally symmetric equation—such as the other master equations we studied above. Of course, this result can be used to derive closed-form solutions in other models with a maximally symmetric master equation.

Recall that a map in the space of the variables which transforms one solution to any other solution was also found in [57]. However, while both maps transform solutions into solutions, the one that we have discussed here, transforms not only solutions into solutions but systems of dynamical equations into equivalent systems^3^. In order to reflect that latter property, the map is called an equivalent point transformation.

The elements of the $SL\left( 3,R\right)$—except for the transformations which relate algebraic equivalent equations— provide us with important physical information about the system under study. One of these properties which arises from eq. (26) is the well-known scale invariance of the Harrison–Zeldovich spectrum, regarding which it can easily be seen that equation (26) is invariant under transformations $\omega =\omega ^{\prime }+\omega _{0}$ or $\omega =\omega ^{\prime }e^{{\bar{\omega }}_{0}}$, where these two transformations are related with the symmetry vectors $\partial _{\omega }$ and $\omega \partial _{\omega }$. In particular, every element of the $SL\left( 3,R\right)$ is related to a point transformation which leaves the differential equation, and consequently the solution, invariant. Moreover, with a different reparameterization of the $SL\left( 3,R\right)$, for equivalent models, the physical interpretation of the invariant point transformations can change between the different models.

## Conclusions

In scalar-field cosmology, the dark-energy EoS and the inflationary scalar-field potential have been reconstructed from the spectral index, $n_{s}$. From the Planck 2015 data analysis, it is known that the observable variables—the tensor-to-scalar ratio, *r*, and the spectral index for the density perturbations, $n_{s}$—form a surface in the $n_{s}-r$ plane. Furthermore, these two observable variables can be expressed in terms of the slow-roll parameters and their derivatives. Therefore, the ansatz that the spectral index for the density perturbations is related with the tensor-to-scalar ratio, $\left( n_{s}-1\right) =h\left( r\right)$, provides a differential (master) equation whose solution defines the corresponding cosmological model.

In this paper, we assumed $n_{s}$ to be given in the first approximation by a function $h\left( r\right)$ that it is: (a) constant, (b) linear, and (c) quadratic, respectively. In order for the first-order approximation to be valid the free parameters which have been introduced by the function $h\left( r\right)$ have to satisfy some consistency conditions.

We work with the HSR parameters. The case in which $h\left( r\right)$ is constant, that is, $n_{s}-1=-2n_{0},\,$ is one that has been studied before in the literature and, in the limit, $n_{0}=0$, corresponds to the Harrison–Zeldovich spectrum. The differential equation which follows provides the scalar factor to be that of a specific intermediate inflation, $a\left( t\right) \simeq \exp \left( a_{1}t^{2/3}\right)$, while the corresponding perfect fluid satisfies the equation of state (28). On the other hand, for nonzero $n_{0}$, we found that the scalar field satisfies an EoS given by expression (48) for $\lambda =2,$ which includes expression (28). For the scalar-field potential, the construction looks similar, and for $n_{0}=0$ the potential is given in terms of polynomials of the field $\phi$, and for $n_{0}\ne 0$ in terms of hyperbolic trigonometric functions.

As a second generalization, we assumed $h\left( r\right)$ to be the linear function, $h\left( r\right) =-\frac{n_{1}}{5}r-2n_{0}$. Now, the models derived from the differential equation $n-1=h\left( r\right) ,$ in the first-order approximation, are the generalized Chaplygin gases, (28) and (48), for $n_{0}=0$ and $n_{0}\ne 0,$ respectively; where now the power $\lambda$ in the equations of state is related to the value of $n_{1}$, by $\lambda =2-n_{1}$.

Finally, the case in which $h\left( r\right)$ is a quadratic polynomial was considered and two new equations of state which generalize the Chaplygin gas were derived. Exact examples displaying a generalised sudden singularity of the type identified by Barrow and Graham [62] for inflationary scalar fields with fractional potentials were found here. Lastly, the ranges for the values of the free parameters of the models have been considered which permit the universe to escape from the inflationary phase.

It is important to mention that in this work we have assumed that we are in the inflationary epoch and so the equation of state parameters, or equivalently the scalar field potentials that we reconstructed, can be seen as the leading order terms, or attractors, of a more general equation of state parameter which describes the whole evolution of the universe.

It is particularly interesting that the master equations we derived in our study are second-order differential equations of maximal symmetry. Hence, they are invariant under the action of point transformations with generators given by the elements of the $SL\left( 3,R\right)$ algebra. Every master equation defines a representation of the $SL\left( 3,R\right)$ algebra and the map which changes the representation transforms the master equation to the corresponding master equation of another model. This relates explicitly the form of the line elements for the various cosmological models. The transformation which performs the change is a projective transformation in the jet-space of the master equation; that is, a map in the space of the dependent variable $F\left( \omega \right)$ and the spacetime variable $\omega$ – we recall that $dt=e^{-F\left( \omega \right) /2}d\omega$ and $a\left( t\right) =e^{\omega /6}$.

In a forthcoming work we will investigate whether the latter result can be extended to the case in which the master equation, $n_{s}-1=h\left( r\right)$, is defined by higher-order approximations for the spectral indices.

## Footnotes

1 Where a prime “$^{\prime }$” denotes the total derivative with respect to $\omega$.

2 According to Lie’s Theorem, any second-order equation which admits the elements of the $SL\left( 3,R\right)$ algebra as symmetries is equivalent to the equation of a free particle and all the maximally symmetric equations commute [58]. The map is the one which transforms the admitted $SL\left( 3,R\right)$ Lie algebra among the different representations of the admitted equations, for more details see [59].

3 Other transformations which belong to these families of transformations are presented in [18].
